# Molecular epidemiology of mumps viruses in the Netherlands, 2017-2019

**DOI:** 10.1371/journal.pone.0233143

**Published:** 2020-09-14

**Authors:** Rogier Bodewes, Linda Reijnen, Jeroen Kerkhof, Jeroen Cremer, Dennis Schmitz, Rob van Binnendijk, Irene K. Veldhuijzen

**Affiliations:** Centre for Infectious Disease Control, National Institute for Public Health and the Environment (RIVM), Bilthoven, The Netherlands; Universidade Federal do Rio de Janeiro, BRAZIL

## Abstract

Mumps cases continue to occur, also in countries with a relatively high vaccination rate. The last major outbreaks of mumps in the Netherlands were in 2009–2012 and thereafter, only small clusters and single cases were reported. Molecular epidemiology can provide insights in the circulation of mumps viruses. The aims of the present study were to analyze the molecular epidemiology of mumps viruses in the Netherlands in 2017–2019 and to compare the phylogenetic trees built from sequence data of near complete mumps virus genomes or from the SH gene and non-coding regions (SH+NCRs). To this end, Sanger sequence data from SH+NCRs were analyzed from 82 mumps genotype G viruses. In addition, near complete genomes were obtained from 10 mumps virus isolates using next-generation sequencing. Analysis of SH+NCRs sequences of mumps genotype G viruses revealed the presence of two major genetic lineages in the Netherlands, which was confirmed by analysis of near complete genomes. Comparison of phylogenetic trees built with SH+NCRs or near complete genomes indicated that the topology was similar, while somewhat longer branches were present in the phylogenetic tree with near complete genomes. These results confirm that analysis of SH + NCRs sequence data is a useful approach for molecular surveillance. Furthermore, data from recent mumps genotype G viruses might indicate (intermittent) circulation of mumps genotype G viruses in the Netherlands in 2017–2019.

## Introduction

Mumps viruses are single stranded negative-sense RNA viruses belonging to the genus *Orthorubulavirus* of the family of *Paramyxoviridae*. Infection of humans with mumps virus results in acute illness which is usually characterized by a temporary unilateral or bilateral parotitis. Occasionally, mumps virus infections can result in serious complications [1, 2], but infections with mumps virus also often do not result in recognized clinical signs [1, 3].

In 1987, vaccination against mumps virus was implemented in the National Immunization Program in the Netherlands. Although this resulted in a rapid decline of mumps cases, in recent years multiple outbreaks occurred mainly among vaccinated young adults [4, 5]. The last major outbreaks of mumps in the Netherlands were in 2009–2012 [5]. Thereafter, only small clusters and single cases were reported [6]. From 2013 until 2020, multiple small local outbreaks and individual mumps cases were reported in the Netherlands [6].

Molecular epidemiology can provide insights in the circulation of mumps viruses. Sequencing of the SH gene and adjacent non-coding regions provides information about the genotype and some information of circulating strains [7, 8]. Increasing the molecular resolution by obtaining additional sequence data of mumps viruses has proven useful in defining different transmission chains and local clusters [7, 9, 10]. In this context, sequencing of the hemagglutinin-neuraminidase protein gene (HN) and fusion protein gene (F) provides similar sequence resolution compared to sequencing of the sum of three non-coding regions (NCRs), present between the nucleocapsid protein and phosphoprotein (N-P), between phosphoprotein and matrix protein (P-M) and between matrix protein gene and F gene (M-F). However, sequencing of the complete genome might provide the more complete resolution that is necessary to identify exact transmission chains and it has the advantage of easy comparison with other mumps viruses on a global scale [11–15].

The aims of the present study were to analyze the molecular epidemiology of mumps viruses in the Netherlands in 2017–2019 and to compare phylogenetic trees built using near complete genomes with SH+NCRs. To this end, we investigated all typable specimens generated from isolated viruses of molecular confirmed mumps cases in the Netherlands from 2017 to 2019.

## Materials and methods

### Sample collection, molecular detection, Sanger sequencing and virus isolation

Clinical samples (oral fluid, throat swab and/or urine) from suspected mumps cases (acute unilateral or bilateral parotitis) were submitted by municipal health services or clinical microbiological laboratories in the Netherlands via regular mail at room temperature to the National Institute for Public Health and the Environment (RIVM) for molecular diagnostics. Upon arrival at RIVM, samples were stored at +4°C until further processing. RNA was extracted by automated extraction using the MagNA Pure 96 extraction (Roche Diagnostics) with DNA and Viral NA Small Volume kit and molecular detection of mumps virus RNA was performed by real time quantitative PCR (RT-qPCR) using primers as described previously [16]. In addition, clinical materials from RT-qPCR-confirmed mumps cases, that were notified under the Public Health Act in the Netherlands, were send to the RIVM for molecular surveillance by clinical microbiological laboratories in the Netherlands via regular mail at room temperature. Sequencing of the SH region and NCRs between the N and P, P and M and M and F genes was performed on nucleic acids extracted from clinical materials using the OneStep RT-PCR kit (Qiagen) as described previously [7, 8]. Sanger sequencing was performed at BaseClear (Leiden, the Netherlands). Molecular surveillance of mumps viruses, including this study, is part of the Public Health Act in the Netherlands.

In total 73 clinical materials (oral fluid, throat swabs and urine) were used for virus isolation. Confluent Vero cells (ECACC 84113001) in 24 wells plates (Greiner Bio-One) were inoculated with clinical material (5μl oral fluid, 10μl urine or 50μl throat swab medium) and cells were incubated for one hour at 37°C/5% CO_2_. Subsequently, 1 ml of Dulbecco’s Modified Eagle Medium (DMEM, Gibco) supplemented with 100U/ml Pen-Strep (Lonza), 2 mM L-Glutamine (Lonza), 12.5 μg/ml Amphotericin B (Biowest) and 24 units/ml Nystatin suspension (Sigma) was added and plates were incubated at 37°C/5% CO_2_. Wells were checked daily for the presence of cytopathic effect (CPE). If no CPE was present after five days, cells were trypsinized (0.25% Trypsin-EDTA(1X) Gibco) and subsequently about 1/3 of trypsinized cells were transferred to another well and fresh medium was added. Again, cells were checked daily for the presence of CPE. If no CPE was present for 12 days after the start of the first incubation, culture was considered negative. If CPE was detected, medium was harvested and subsequently passaged one or two times over confluent Vero cells to produce a virus stock in a T25 cm^2^ flask or T75 cm^2^ flask (Corning) resulting in 8 or 25 ml cell culture supernatant medium containing mumps virus.

### Next-generation sequencing

Mumps virus isolates were processed for full genome sequencing using a viral metagenomics approach. To this end, 200μl of each cell culture supernatant was pretreated and processed for next-generation sequencing using the Illumina NextSeq 550 platform essentially as described elsewhere [17](Benschop et al, manuscript submitted). In brief, cDNA was prepared from pre-treated and extracted RNA from cell culture supernatant. Subsequently, double stranded DNA was synthesized. Double stranded DNA was purified and concentrated and used to prepare libraries using the Nextera XT DNA Library Prep Kit (Illumina). Sequencing was performed on the NextSeq with the NextSeq 500/550 Mid Output Kit v2.5 (300 Cycles). Bioinformatic analysis of obtained reads was performed using the Jovian pipeline with default parameters (Schmitz *et al*, manuscript in prep; https://github.com/DennisSchmitz/Jovian).

### Genetic and phylogenetic analysis

Mumps virus genotypes were determined based on SH sequences obtained from detected mumps viruses by comparison with reference strains using BioNumerics version 7.6.3 [18–20]. Genotyping was confirmed by basic local alignment search tool (BLAST) analysis [21, 22].

Obtained sequence data were aligned manually in MEGA7 and phylogenetic trees were constructed using the maximum likelihood method with IQ-tree software via the webserver (W-IQ-TREE) [23–25]. For each alignment, the best-fit substitution model according to bayesian information criterion (BIC) was selected based on analysis with ModelFinder [26]. Branch support in the different phylogenetic trees was calculated using the ultrafast bootstrap approach (UFboot) with 1000 bootstrap alignments [27]. Phylogenetic trees were visualized using FigTree v1.4.4 [28]. Complete and/or partial sequence data from mumps viruses mumps virus genotype C (AY669145), Mumps virus Jeryl-Lynn (AF338106), Mumps virus strain 9218/Zg98 (EU370206), MuV/California.USA/50.07/1 (JX287386), MuV/DU.CR/O05 (EU370207), MuV/Iowa.USA/06/6 (JX287385), MuV/New York.USA/01.10 (JX287389), MuV/New York.USA/40.09 (JX287390), MuV/New York.USA/53.09/3 (JX287387), MuVi.Ontario.CAN/04.10 (KY006857), MuVi.Ontario.CAN/38.09/2 (KY680537), MuVi/Chennai.IND/35.12 (KF843896), MuVi/Dadra&NagarHaveli.IND/42.16/2 (MG460606), MuVi/Davangere.IND/01.14 (KX953297), MuVi/Gloucester.GBR/32.96 (AF280799), MuVi/Kushinagar.IND/36.13 (KM385447), MuVi/London.GBR/03.02 (KF878082), MuVi/NewPlymouth.NZL/30.17 (MG765426), MuVi/Ontario.CAN/13.10 (KY006858), MuVi/Ontario.CAN/37.09 (KY006856), MuVi/Ontario.CAN/40.09/1 (KY680538), MuVi/Osmanabad.IND/10.12/17 (KF843894), MuVi/Osmanabad.IND/10.12/18 (KF843895), MuVi/Pune.IND/00.86 (KF738113), MuVi/Pune.IND/07.12 (KF738114), MuVi/Pune.IND/50.08 (KF843893), MuVi/RW154.USA/0.70s (KF878080), MuVi/Split.CRO/05.11 (JN635498), MuVi/Springdale_754/2016 (KY996512), MuVi/Zagreb.HRV/28.12 (KF481689), MuVs/Arkansas.USA/43.16/[G] isolate 20171006/BC06 (MK585579), MuVs/Illinois.USA./26.15/2 (MG986460), MuVs/Indiana.USA/48.16 (MG986406), MuVs/Massachusetts.USA/21.16 (MF965260), MuVs/Massachusetts.USA/24.17/5 (MG986430), MuVs/Massachusetts.USA/26.15 (MF965222), MuVs/Massachusetts.USA/30.16 (MF965299), MuVs/Massachusetts.USA/37.15 (MF965262), MuVs/Massachusetts.USA/37.16 (MF965284), MuVs/Michigan.USA/4.16 (MG986419), MuVs/Montana.USA/11.16 (MG986420), MuVs/Ohio.USA/11.14 (MG986426), and MuVs/Pennsylvania.USA/19.16 (MG986458), were included in the alignments as reference sequences and to allow comparisons between mumps viruses detected in other countries and the Netherlands. Selection of these strains was based on BLAST analysis of obtained mumps virus near complete genomes and a manual selection of complete genomes of mumps genotype G viruses [11–13]. Pairwise identity analyses were performed in MEGA7 using the maximum likelihood method, complete deletion of missing data and otherwise default parameters [23].

## Results

### Mumps cases in 2017, 2018 and 2019 in the Netherlands

Between January 2017 and October 2019, 246 mumps cases were notified in the Netherlands based on clinical symptoms and laboratory confirmation, or an epidemiological link with a laboratory confirmed case. A mumps virus genotype could be determined for 123 cases (50%), of which 108 cases (88%) belonged to genotype G. The other 15 cases belonged to either genotype C (five cases), D (one case), H (seven cases), J (one case) or K (one case). From in total 82 mumps genotype G cases could NCRs sequence data be obtained in addition to the SH sequences used for genotyping (GenBank accession numbers MT238691-MT238955).

### Virus isolation and next-generation sequencing

Inoculation of nine throat swabs and one oral fluid specimen (14%) resulted in a mumps virus isolate as determined by the presence of cytopathic effect. Eight mumps virus isolates belonged to genotype G, while the other two mumps virus isolates belonged to genotype C or K (Table 1). Near complete genomes (excluding 3’ and 5’ termini) were obtained from all isolates, with genome sizes ranging from 15144 to 15375 nucleotides (GenBank accession numbers MT238681-MT238690, European Nucleotide Archive numbers ERS4582384-90).

**Table 1:** Overview of metadata of mumps virus isolates.

Isolate	Clinical specimen	Epidemiological link	Genotype	Mean read coverage*	GenBank accession number	Genome size	Location^#^ of nucleotide differences between clinical material and isolate
MuVi/Amsterdam.NLD/29.17/1	Throat swab	Southern Europe, travelling.	G	3200	MT238681	15361	2141A>C
MuVi/Beugen.NLD/16.18	Throat swab	The Netherlands, unknown.	G	4300	MT238682	15284	not detected
MuVi/Eindhoven.NLD/42.18	Oral fluid	The Netherlands, cluster among students at a university.	G	550	MT238684	15145	not detected
MuVi/Utrecht.NLD/8.19	Throat swab	Southeast Asia, travelling.	K	2000	MT238690	15363	not detected
MuVi/Noord-Holland.NLD/9.19	Throat swab	The Netherlands, unknown.	G	2500	MT238686	15365	not detected
MuVi/Deventer.NLD/12.19	Throat swab	The Netherlands, cluster among students at a university.	G	2300	MT238683	15375	6413T>C
MuVi/Utrecht.NLD/15.19	Throat swab	The Netherlands, contact with students at an university, possibly same cluster as MuVi/Deventer.NLD/12.19.	G	480	MT238687	15375	not detected
MuVi/Utrecht.NLD/21.19	Throat swab	East-Asia, travelling.	C	1400	MT238689	15369	not detected
MuVi/Gelderland.NLD/26.19	Throat swab	The Netherlands, work.	G	3700	MT238685	15364	not detected
MuVi/Utrecht.NLD/35.19	Throat swab	The Netherland, unknown.	G	2600	MT238688	15175	2924A/G>G

*if the sequence consisted of two or more overlapping contigs by de-novo assembly, the mean coverage of the contig with the lowest coverage is indicated.

#location was based on the position in the complete genome of Mumps virus (strain Jeryl-Lynn) live major vaccine component (GenBank accession number AF338106).

### Comparisons between sequence data of original materials and isolates by Sanger sequencing

Comparison of nucleotide sequence data of SH+NCRs obtained from mumps viruses detected in clinical materials by Sanger sequencing with consensus sequence data from isolates obtained by NGS revealed in total three differences. At position 144 of the SH gene of MuVi/Deventer.NLD/12.19 a C was present (corresponding to position 6413 of the complete genome of Mumps virus -strain Jeryl-Lynn- live major vaccine component), while a T was detected at that position in the original material of MuVs/Deventer.NLD/12.19. Sanger sequencing confirmed the presence of a C at this position in the isolate. Additional Sanger sequencing revealed that the nucleotide shift from T to C was already present after the first passage.

At position 443 of the N-P NCR (corresponding to position 2141 of the complete genome of Mumps virus -strain Jeryl-Lynn- live major vaccine component), a C was detected in the sequence data from MuVi/Amsterdam.NLD/29.17/1, while an A was detected in the sequence data obtained from the original material (MuVs/Amsterdam.NLD/29.17/1). Since this nucleotide position was in the phosphoprotein gene, this nucleotide change resulted also in an amino acid change (lysine to glutamine). The presence of the C in the isolate was confirmed by Sanger sequencing. Additional Sanger sequencing analyses of the various passages indicated that the C was already detected at this position as a minor variant after the first passage, while two peaks of equal size were detected after the second passage. In the P-M NCR of MuVs/Utrecht.NLD/35.19, two peaks of similar size (A/G) were detected in the Sanger sequence data (both forward and reverse sequence) at position 76 (corresponding to position 2924 of the complete genome of Mumps virus -strain Jeryl-Lynn- live major vaccine component), while the consensus sequence of MuVi/Utrecht.NLD/35.19 was a G at that position. This nucleotide position was also in the phosphoprotein, but this nucleotide difference did not result in an amino acid change. Additional analysis of the first passage and second passage of this isolate by Sanger sequencing indicated that in all these passages both nucleotide variants were present with two peaks of equal size. No other nucleotide differences were detected between the sequence data obtained from the original materials and the isolates.

### Phylogenetic analysis of concatenated SH+NCRs sequences

**Fig 1:**
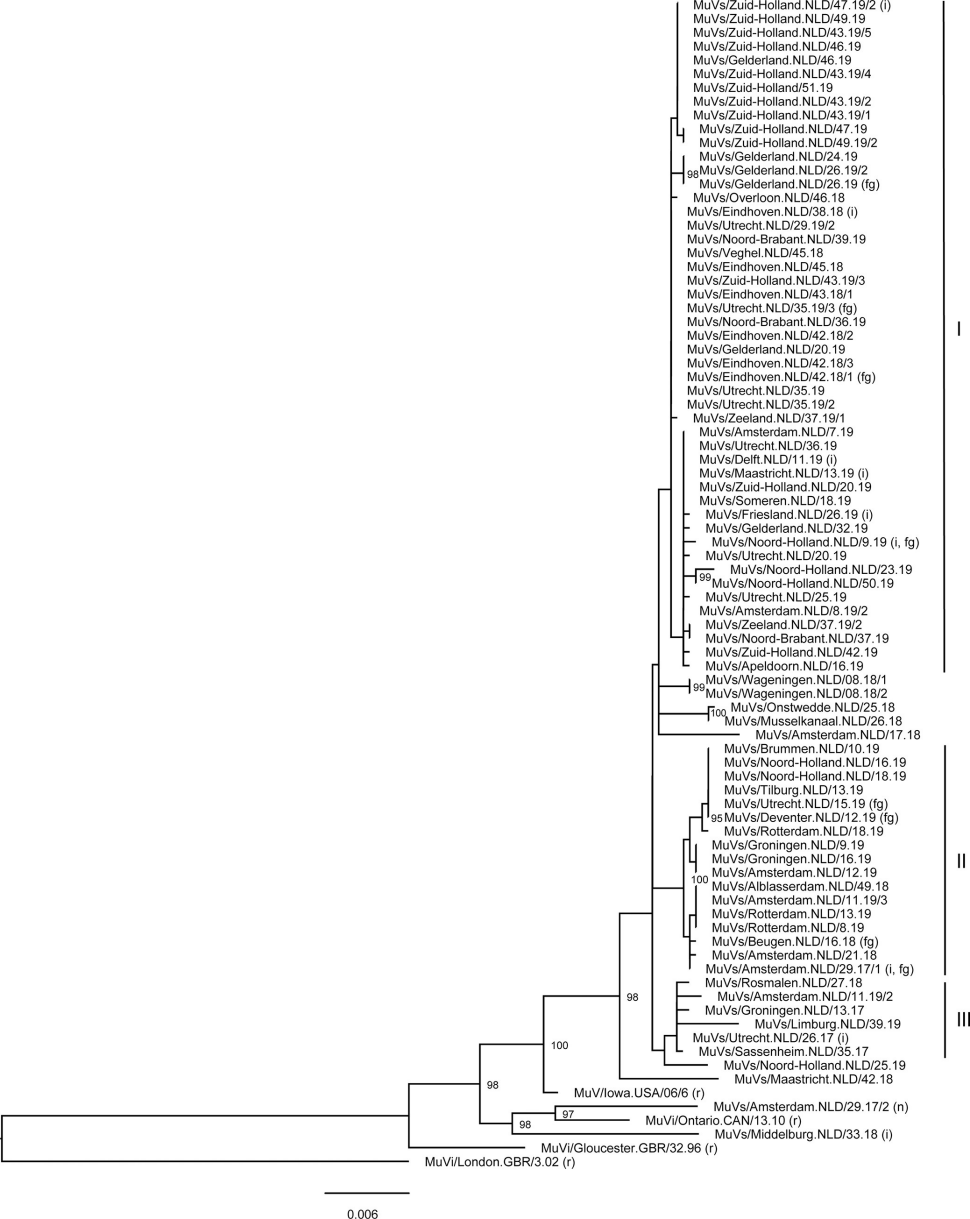
Phylogenetic analysis of concatenated sequence data of SH and NCRs of mumps viruses detected in the Netherlands from 2017–2019 using the maximum likelihood method. For this phylogenetic analysis, the transition model (TIM) +F+I was the best-fit model according to BIC. Only bootstrap values ≥95 are indicated. The phylogenetic tree was rooted using MuVi/London.GBR/3.02. The main genetic lineages of mumps viruses detected in the Netherlands are indicated with I, II and III, while mumps viruses collected from cases from which it was known that they were not infected in the Netherlands are indicated with ‘i’ (import) behind the virus name. Viruses from which also near complete genomes were obtained after isolation, are indicated with ‘fg’, while reference viruses are indicated with ‘r’.

**Fig 2:**
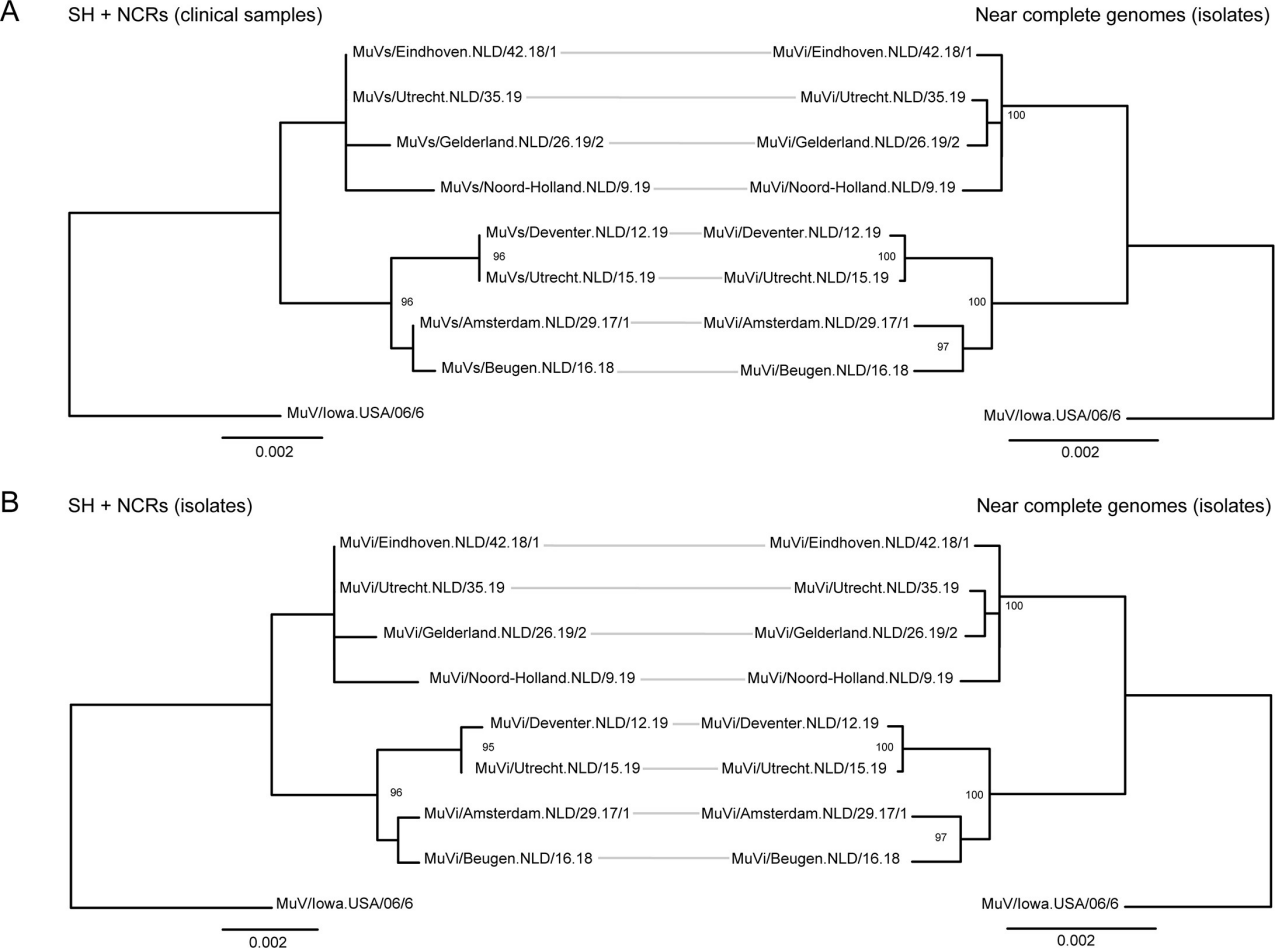
Comparison of phylogenetic topology obtained by analysis of near complete genomes and SH+NCRs. Phylogenetic analysis was performed on near complete genomes of isolates of mumps genotype G viruses and results were compared with SH+NCRs data obtained from the same set of mumps viruses detected in clinical materials (A) and in isolates (B). Phylogenetic trees were built with the maximum likelihood method and the generable time reversible (GTR)+ F+G4 model was used based on selection as the best-fit model according to BIC for the analysis of near complete genomes. Both trees were rooted using MuV/Iowa.USA/06/6. Only bootstrap values ≥95 are indicated.

Phylogenetic analysis of concatenated SH+NCRs mumps genotype G virus sequences indicated that two main lineages of genotype G virus strains were detected in the Netherlands in 2017–2019 with respectively 49 and 17 mumps viruses (Fig 1), which differed by in total eight nucleotides in the SH and NCRs. Viruses of lineage I were only detected in the second half of 2018 and 2019, while viruses from lineage II were detected in 2017, 2018 and 2019. Another lineage of mumps genotype G viruses consisted of six cases, but no groups of viruses with identical sequence were detected within this lineage (lineage III). The presence of two main lineages was confirmed by analysis of near complete genomes of mumps viruses detected in these cases (Fig 2).

Within these three lineages, multiple sequence variants were detected in the Netherlands. In addition, various other virus variants were detected in the Netherlands in 2017, 2018 and 2019, but these variants were only detected in a limited amount of cases. Based on epidemiological data, 69 mumps viruses included in this phylogenetic analysis were detected in cases that were infected in the Netherlands. From 9 cases, it was known that they were infected in another country, while the country of infection was unclear in 3 mumps cases. Mumps viruses detected in 6 from these 9 cases that were not infected in the Netherlands belonged to lineage I, while the other three mumps viruses belonged to either lineage II (one mumps virus), lineage III (one mumps virus), or one of the viruses that were not part of a lineage (one mumps virus) (Fig 1).

### Comparison of genetic differences and phylogenetic topology between SH+NCRs and near complete genomes

Comparison of the genetic differences provided by concatenated SH+NCRs sequences and near complete genomes revealed an increase in the number of nucleotide differences if near complete genomes were analyzed. For example, SH+NCRs sequences of MuVs/Utrecht.NLD/35.19 and MuVs/Eindhoven.NLD/42.18/1 were identical, while 11 nucleotide differences were detected by analysis of near complete genomes. The pairwise identities for these two viruses were 100% for the SH+NCRs and 99.97% for the near complete genomes. In addition, one nucleotide difference was present between the SH+NCRs sequences of MuVs/Amsterdam.NLD/29.17/1 and MuVs/Beugen.NLD/16.18, while 16 nucleotide differences were present between the isolated near complete genomes of these two viruses, although one of these differences was confirmed to be related to isolation of the virus. Pairwise identities between the isolates of these viruses were 99.91% for the SH+NCRs and 99.87% for the near complete genomes. Furthermore, MuVs/Deventer.NLD/12.19 and MuVs/Utrecht.NLD/15.19 were identical for SH+NCRs sequences, while four nucleotide differences were present between the near complete genomes of these isolated viruses (including one related to isolation of the virus), resulting in pairwise identities of 99.96% for the SH+NCRs and 99.97% for the near complete genomes (Fig 2). The average pairwise identity between the eight mumps virus isolates was 99.60% for the SH+NCRs and 99.66% for the near complete genomes.

Comparison of the phylogenetic topology between SH+NCRs and near complete genomes indicated that a similar branching pattern was observed in both phylogenetic trees, except for the branching pattern of MuVi/Utrecht.NLD/35.19 and MuVi/Gelderland.NLD/26.19/2. The two nucleotide changes in SH+NCRs of MuVi/Deventer.NLD/12.19 and MuVi/Amsterdam.NLD/29.17/1 compared to clinical samples resulted in increased branch lengths, but did not change the phylogenetic topology (Fig 2).

### Phylogenetic analysis of near complete mumps viruses

**Fig 3:**
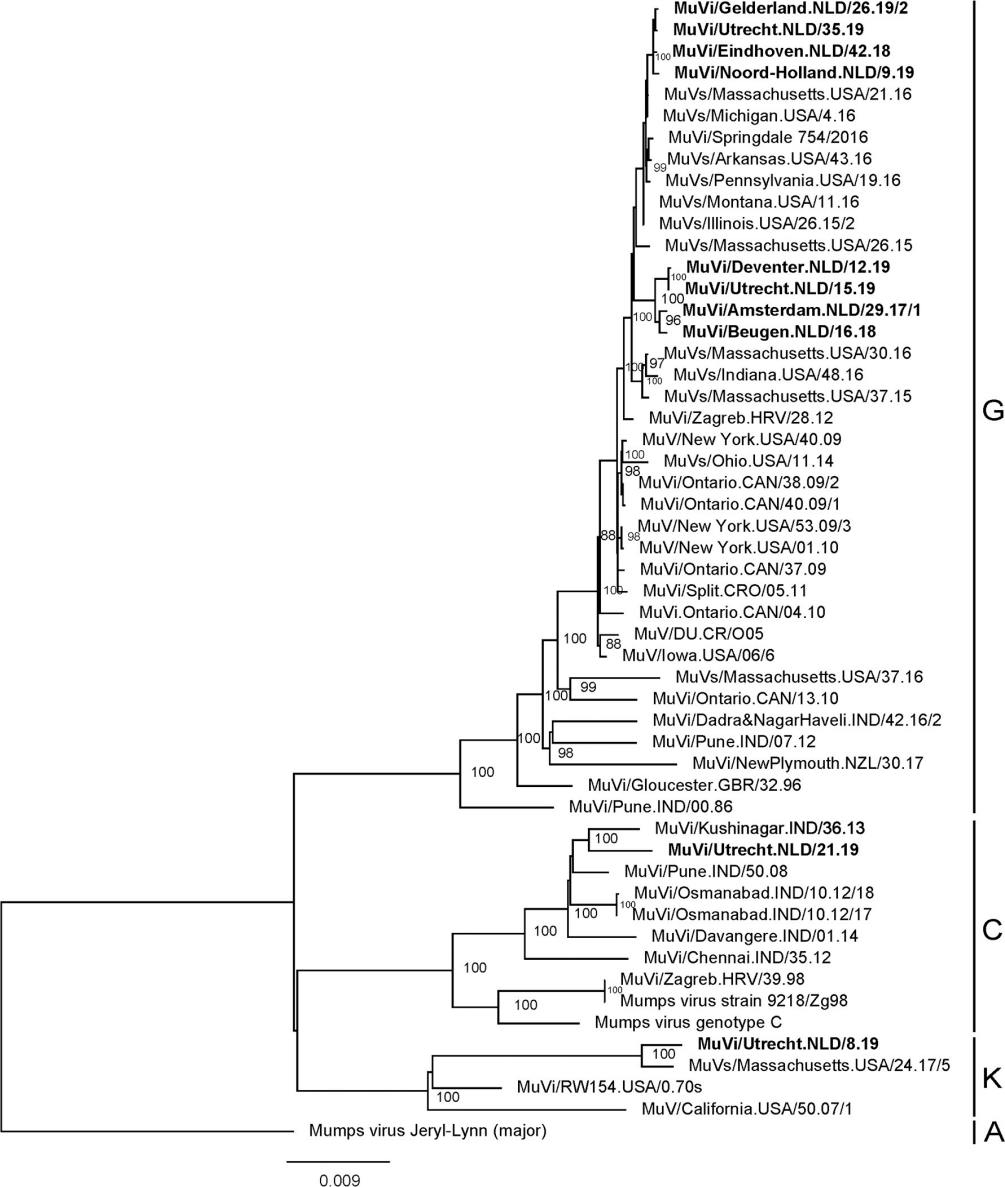
Phylogenetic analysis of complete mumps virus genomes. Phylogenetic analysis was performed on near complete genomes (excluding 3’ and 5’ termini) of mumps virus isolates detected in the Netherlands and various closely related and representative mumps viruses using GTR+F+G4 model based on analysis using ModelFinder [26]. Only bootstrap values ≥95 are indicated. Mumps viruses detected in the Netherlands are indicated in bold. Genotypes to which these viruses belong are indicated and the tree was rooted using Mumps virus Jeryl-Lynn (major strain).

Alignment and subsequent phylogenetic analysis of the near complete genomes (excluding 3’ and 5’ termini) obtained from mumps virus isolates detected in the Netherlands with several representative and closely related mumps viruses indicated that these viruses also belonged to two separate groups of viruses (Fig 3). Pairwise identity analysis on the nucleotide level of mumps genotype G viruses of the near complete genomes of MuVi/Gelderland.NLD/26.19/2, MuVi/Utrecht.NLD/35.19, MuVi/Eindhoven.NLD/42.18 and MuVi/Noord-Holland.NLD/9.19 confirmed that these viruses were most closely related to MuVs/Michigan.USA/4.16 (99.90–99.91%) and MuVs/Massachusetts.USA/21.16 (99.56–99.88%). MuVi/Amsterdam.NLD/29.17/1, MuVi/Beugen.NLD/16.18, MuVi/Deventer.NLD/12.19 and MuVi/Utrecht.NLD/15.19 were most closely related to MuVs/Montana.USA/11.16 and MuVs/Illinois.USA/26.15/2 (99.59–99.63%). Mumps virus genotype K virus MuVi/Utrecht.NLD/8.19 was most closely related to MuVs/Massachusetts.USA/24.17/5[K] (99.37%) and mumps virus genotype C virus MuVi/Utrecht.NLD/21.19 was most closely related to MuVi/Kushinagar.IND/36/13[C] (99.01%) (Fig 3).

## Discussion

Mumps cases continue to occur, also in countries with a relatively high vaccination rate. Molecular epidemiology could contribute to the understanding of mumps virus circulation. In the present study, the molecular epidemiology of mumps virus was evaluated using SH+NCRs mumps virus sequences obtained from mumps cases in the Netherlands in 2017–2019. Analysis of these regions with the relatively highest variation of the mumps virus genome revealed the presence of two major lineages in the Netherlands. The presence of these two lineages was confirmed by analysis of near complete genomes of a selection of mumps viruses.

The presence of two lineages with multiple closely related mumps viruses in recent years, might indicate that there was (intermittent) circulation of mumps viruses in the Netherlands in recent years. This is supported by the fact that near complete genomes of both lineages are distinct from mumps viruses detected recently in mainly the United States of America and Canada [11, 29]. Of interest, viruses of lineage I were not detected before the second part of 2018, suggesting that this lineage emerged recently in the Netherlands. However, recent SH+NCRs mumps virus sequences from nearby countries were not available and therefore it is not possible to draw conclusions. In addition to mumps genotype G viruses, mumps viruses were detected with other mumps genotypes. Since these mumps viruses were detected only in a relatively small proportion, these viruses were most likely not endemic in the Netherlands in recent years.

Of interest, comparison of SH+NCRs sequence data from original materials with that of isolates indicated that passaging of mumps viruses over cells resulted in three nucleotide differences. Since the sequence of 2270 nucleotides of in 10 total viruses were compared, it was calculated that 0.013% of all nucleotide positions changed due to passaging (15384*0.00013 = ~2 nucleotide changes for a full mumps virus genome). Therefore, sequence data from isolates should be interpreted carefully, especially if used to understand exact transmission trees. To study transmission trees, preferably complete genomes obtained from original materials or eventually viruses that are passaged only a single time over cells are used [11, 29].

Most mumps viruses from which a near complete genome was obtained in this study were collected from mumps cases that occurred several months after each other, and therefore it cannot be concluded whether these viruses are part of the same transmission chain. Of interest, mumps viruses MuVi/Noord-Holland.NLD/9.19, MuVi/Deventer.NLD/12.19 and MuVi/Utrecht.NLD/15.19 were collected within six weeks. While the sequence of MuVi/Noord-Holland.NLD/9.19 was relatively distinct from the other two viruses, between MuVi/Utrecht.NLD/15.19 and MuVi/Deventer.NLD/12.19 were only four nucleotide differences present. Since both mumps cases also lived in the same city, they were most likely part of the same mumps cluster, but there was no direct epidemiological link between these two cases.

Comparison of phylogenetic trees prepared by analysis of SH+NCRs and near complete genomes indicated that the topologies of both trees were similar, while branches lengths were different. Furthermore, the mean pairwise identity of the SH+NCRs regions was slightly lower than the mean pairwise identity of the near complete genomes (99.60% vs. 99.66%), which confirms that the SH+NCRs regions are relatively variable regions of the mumps virus genome [18]. Therefore, analysis of SH+NCRs sequences is a useful approach for molecular surveillance [7, 8]. However, to study exact transmissions trees, preferably complete genomes are analyzed [11, 29].

In conclusion, while in recent years no major outbreaks of mumps occurred in the Netherlands, the presence of two main lineages of multiple mumps genotype G viruses with identical or very similar SH+NCRs sequences might indicate (intermittent) circulation of mumps viruses in the Netherlands in 2017–2019.
